# Nanoparticle applications in agriculture: overview and response of plant-associated microorganisms

**DOI:** 10.3389/fmicb.2024.1354440

**Published:** 2024-03-06

**Authors:** Katiso Mgadi, Busiswa Ndaba, Ashira Roopnarain, Haripriya Rama, Rasheed Adeleke

**Affiliations:** ^1^Unit of Environmental Sciences and Management, North-West University, Potchefstroom, South Africa; ^2^Microbiology and Environmental Biotechnology Research Group, Agricultural Research Council-Natural Resources and Engineering, Pretoria, South Africa; ^3^Department of Environmental Sciences, University of South Africa–Florida Campus, Johannesburg, South Africa; ^4^Department of Physics, University of South Africa–Florida Campus, Johannesburg, South Africa

**Keywords:** agriculture, rhizosphere, endosphere, nanoparticles, microorganisms

## Abstract

Globally, food security has become a critical concern due to the rise in human population and the current climate change crisis. Usage of conventional agrochemicals to maximize crop yields has resulted in the degradation of fertile soil, environmental pollution as well as human and agroecosystem health risks. Nanotechnology in agriculture is a fast-emerging and new area of research explored to improve crop productivity and nutrient-use efficiency using nano-sized agrochemicals at lower doses than conventional agrochemicals. Nanoparticles in agriculture are applied as nanofertilizers and/or nanopesticides. Positive results have been observed in terms of plant growth when using nano-based agricultural amendments. However, their continuous application may have adverse effects on plant-associated rhizospheric and endospheric microorganisms which often play a crucial role in plant growth, nutrient uptake, and disease prevention. While research shows that the application of nanoparticles has the potential to improve plant growth and yield, their effect on the diversity and function of plant-associated microorganisms remains under-explored. This review provides an overview of plant-associated microorganisms and their functions. Additionally, it highlights the response of plant-associated microorganisms to nanoparticle application and provides insight into areas of research required to promote sustainable and precision agricultural practices that incorporate nanofertilizers and nanopesticides.

## 1 Introduction

Food security is currently a global concern due to the exponential growth of human population coupled with the ongoing climate crisis. The conventional approach for improvement of crop productivity to sustain the growing population is by applying bulk chemical fertilizers (congruent with terms synthetic, inorganic, and mineral fertilizers) and pesticides. However, there is evidence that only a fraction of the chemical fertilizers and pesticides applied contribute to aiding crop production (Raliya et al., 2018; Tudi et al., 2021). Unfortunately, their residues, to a great extent, pollute the environment and groundwater via leaching. These pollutants cause soil degradation that could be in form of acidification and eutrophication. Such processes are usually hazardous to aquatic and agroecosystems (Tudi et al., 2021).

In spite of the potential yield benefits associated with bulk chemical fertilization, it is capable of changing the chemical properties of the soil and does not improve the richness, diversity, or abundance of soil microbial communities, which are generally indicators of fertile soil (Dincǎ et al., 2022). The viability and metabolic activity of bacterial and fungal species in the soil were reported to be impacted by excessive concentrations of chemical fertilizers (Dincǎ et al., 2022). On the other hand, pesticides may hinder vital cellular processes of non-target microorganisms and other soil biota which inadvertently results in reduced chemical and biological soil fertility (Vischetti et al., 2020; Tudi et al., 2021).

Recently, the use of nanotechnology in agriculture has been explored as an alternative to the conventional use of bulk chemical fertilizers and pesticides (Kalwani et al., 2022). Nanoparticles can potentially provide various benefits over conventional agricultural practices such as large surface area to volume ratios, mass transfer abilities as well as slow, controlled and targeted delivery of lower nutrient or pesticide concentrations to enhance crop productivity, if used appropriately (Hussain et al., 2023). Although the application of nanoparticles has been revolutionary for crop productivity, the response of plant-associated microorganisms to nano-based amendments remains unclear. Similarly to bulk chemical fertilizers or pesticides, nano-based agricultural amendments may have an impact on plant-associated microorganisms. Exposure of plant-associated microorganisms to nanoparticles can either be beneficial or harmful depending on various factors. Hence, further investigation on the interaction and response of plant-associated microorganisms to nanoparticles is warranted to ensure sustainable precision agricultural practices.

Microorganisms are extremely important for overall soil and plant health (Goswami et al., 2016). Diverse genera of microorganisms have been identified in soils and crops, and they play an important role in the regulation of agroecosystem productivity, soil physicochemical characteristics, and plant health (Dincǎ et al., 2022). A vast majority of plant-associated microorganisms are found in the soil, near plant roots in the region called the rhizosphere, where they serve essential ecological functions such as promoting plant growth (Bello-Akinosho et al., 2021). Although plants associated microorganisms can exert both negative and postive impacts of on the host plants, the focus of this review is majorly on the impact on beneficial plant associated microrogamisms. Plant beneficial bacteria residing in the rhizosphere are termed plant growth-promoting rhizobacteria (PGPR) while microorganisms that colonize the endosphere (interior of the plant) are termed endophytes. Both endophytes and PGPR could be claissfied as Plant growth-promoting microorganisms (PGPMs) if they are able to improve plant growth directly and indirectly. The PGPMs perform crucial metabolic functions which include the decomposition of organic matter, nitrogen fixation, nutrient solubilization, phytohormone production, as well as metabolite synthesis that aids in plant growth and disease prevention (Kalwani et al., 2022).

The diversity and abundance of PGPR in the rhizosphere have been studied since the beginning of the twentieth century (Fagorzi and Mengoni, 2022). However, before the discovery of fungal endophytes in the middle of the twentieth century, the endosphere was long believed to be sterile. Endosphere microbiology, initially dominated by fungal studies, has evolved over the years. Recent development has led to the expansion of the scope with more focus on bacteria. All plant species surveyed to date harbour different microbial communities. Examples of plant microbes that could be beneficial for such relationships are Bacillus and Pseudomonas that have been identified as predominant and diverse genera of PGPR. They play an important role as biocontrol agents through the protection of plants against phytopathogens (Santoyo et al., 2012).

Previous research in plant microbe interactions has laid the foundation by describing certain factors, sometimes referred to as filters, that influence the association. Most notable of these filters are the host plant chemistry, environmental conditions as well as microbe-microbe interactions (Saunders et al., 2010). However, what is not clear is the potential influence of other external factors that are introduced through anthropogenic means linked to technological advancement.

One system in which plant-microbe relationship could be impacted is the use of nanomaterials in agro-ecosystem. Applications of nanomaterials are becoming increasingly popular, especially with their usage as nanofertilizers and nanopesticides for precision and sustainable agriculture. Unfortunately, there is no existing guideline that reflects their potential impacts on plant associated microbes. Hence, this review aims to provide insights about existing literatures on this topic and reflects on potential implication for sustainable agricultural practices.

## 2 Plant-associated microorganisms in agriculture

The survival of the plant is greatly dependent on its plant-associated microorganisms (Backer et al., 2018). Similarly, the diversity abundance, and actvities of plant-associated microorganisms are affected by the variety of compounds actively released from living plants (exudates). Amino acids, carbohydrates, enzymes, organic acids, hormones, metabolites, and vitamins are examples of plant exudates (Li et al., 2019). This is the community assembly rule driven by the host plant chemistry (Comita et al., 2014). In this instance, plant exert considerable control over the constituents and abundance of plant-associated microorganisms through variations in composition, time of release, and concentration of exudates (Backer et al., 2018; Adeleke et al., 2019). By providing exudates that encourage the growth of specific microorganisms, the plant can shape the plant-associated microbial community (Dlamini et al., 2022). In turn, PGPMs provide plants with various benefits, which include nutrient acquisition, defence against plant pathogens, induction of systemic resistance, and plant growth promotion. Furthermore, PGPMs can protect host plants against abiotic stresses such as salinity, floods, droughts, heavy metal contamination, organic pollutants, and extreme temperatures (Lopes et al., 2021). Currently, much research has been directed to the isolation and characterization of plant-associated microorganisms using omics and sequencing approaches to determine their potential use in agriculture (Trivedi et al., 2021). A summary of the findings of such studies involving PGPR was reported by Saeed et al. (2021). The types of agricultural products consisting of PGPMs with various plant growth-promoting functions are summarized in Table 1. Applications of PGPMs as environmentally-friendly alternatives to agrochemicals involve inoculation of agricultural soils, roots, or seeds. However, the success of microbial inoculation in terms of establishing the PGPMs with persistence for beneficial association with host plants is affected by inoculation methods, environmental conditions, and requirements of host plants and should therefore undergo sufficient research trials prior to specific environmental application (Lopes et al., 2021). Furthermore, climate change and the introduction of emerging alternatives to agrochemicals may influence the productivity of plant holobionts (the plant microbiome and its tissues) (Lopes et al., 2021; Hussain et al., 2023). Hence, it is necessary to investigate the long-term effects of emerging agrochemical alternatives, such as nanoparticles, on plant holobionts. Moreover, future research should investigate the combined use of nanoparticle alternatives and PGPMs that can alleviate abiotic stresses for sustainable crop productivity.

**TABLE 1:** Various applications and functions of plant growth-promoting microorganisms.

Agricultural products	Definition	Plant growth-promoting functions	References
Biofertilizers	Microorganisms or metabolites produced by microorganisms that can be applied to soil, seeds, or plants, to sustain different plant biochemical processes, increase the supply or availability of essential nutrients and/or in some way enhance crop production due to their functions	Phosphate solubilization	Kaur and Purewal (2019)
		Siderophores production	Raimi et al. (2017)
		Exopolysaccharides production	Naseer et al. (2019)
		Biofixation of atmospheric nitrogen	Raimi and Adeleke (2023)
Phytostimulants	Microorganisms with the ability to regulate plant physiology in a beneficial manner through production of secondary metabolites such as ethylene, cytokinins, indole acetic acid (IAA), and gibberellic acid (GA)	Ethylene production	Bano et al. (2022)
		Cytokinins production	
		GA production	
		IAA production	
Biopesticides	Microorganisms or biocontrol agents that control the effects of phytopathogens via the production of metabolites or antibiotics	Hydrolytic enzymes production	Raimi and Adeleke (2023)
		Hydrogen cyanide production	Ajilogba et al. (2022)
		Volatile compounds production	Adeleke et al. (2019)
		Induction of systemic resistance	
		Competition for iron, nutrient and space	
Bioremediators	Microorganisms with the ability to remediate a polluted environment	Siderophores production	Bello-Akinosho et al. (2021)
		Chelate heavy metals	
		Enzyme production for hydrocarbon degradation	Obieze et al. (2022)

### 2.1 Rhizospheric plant-associated microorganisms

**FIGURE 1:**
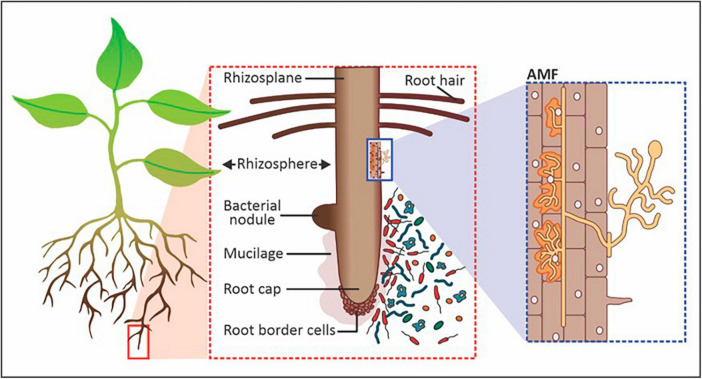
Components of the rhizosphere that includes symbiotic and saprophytic bacteria and arbuscular mycorrhizal fungi (AMF). Image adapted and modified from Philippot et al. (2013).

Microorganisms thrive in the rhizosphere by utilizing root exudates as carbon and nutrient sources for growth and metabolic functions (Dlamini et al., 2022). The width of the rhizosphere ranges from 2 to 80 mm from the root surface, depending on the type of plant (Figure 1). The area of the rhizosphere may expand due to increased exudation, which may be stimulated by increased microbial activity. For instance, mycorrhizal fungi allow plant roots to reach a greater volume of soil through their hyphae while forming a mutualistic symbiotic relationship with the root by obtaining nutrients from and for host plants beyond the rhizosphere (Lanfranco et al., 2017). The plant-associated microorganisms present in and around the rhizosphere function as symbionts, pathogens, as well as food sources for other microorganisms (Munir et al., 2022). The most common genera of PGPR utilized for increasing crop productivity include *Azospirillum*, *Bacillus*, *Burkholderia*, *Enterobacter*, *Flavobacterium*, *Pseudomonas*, *Rhizobium*, *Frankia*, *Clostridium*, *Klebsiella*, *Serratia* and *Streptomyces* (Lopes et al., 2021). In addition to rhizobacteria, fungal groups that play a key role in agricultural productivity include *Aspergillus*, *Fusarium*, *Penicillium*, *Piriformospora*, *Phoma* and *Trichoderma* (Hossain et al., 2017). Of all the interactions between plants and microorganisms in the rhizosphere, biological nitrogen fixation is by far the most researched (Chen et al., 2019; Li et al., 2019; Soumare et al., 2020; Aasfar et al., 2021; Klimasmith and Kent, 2022; Zhang et al., 2023). Nitrogen-fixing bacteria like Rhizobia make unavailable N_2_ accessible to plants. Non-symbiotic or free-living nitrogen-fixing rhizobacteria include *Azotobacter*, *Azospirillum*, *Bacillus*, and *Klebsiella*. Additionally, *Rhizobium*, *Bradyrhizobium*, *Mesorhizobium*, and *Sinorhizobium* collaborate with plants in a symbiotic relationship to exchange nitrogen (N) for growth-promoting nutrients and protection (Munir et al., 2022). Besides nitrogen fixation, PGPMs produce organic chelating compounds that help boost the availability of nutrients like phosphorus (P), manganese (Mn), iron (Fe), zinc (Zn), and copper (Cu) to plants and produce secondary metabolites and phytohormones for biocontrol, plant stimulation and health promotion from the rhizosphere (Emmanuel and Babalola, 2020).

### 2.2 Endospheric plant-associated microorganisms

**FIGURE 2:**
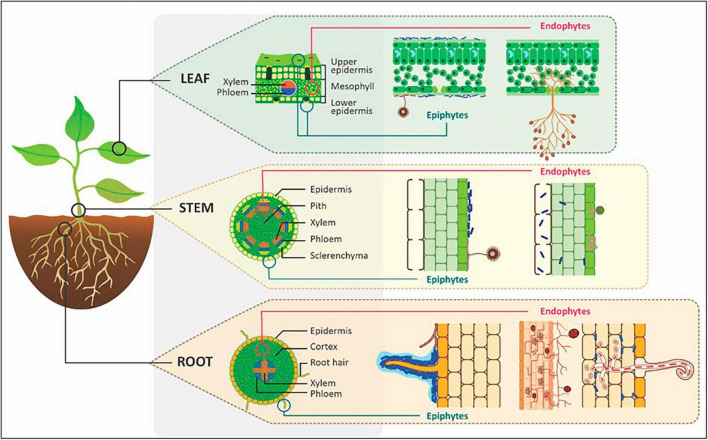
The location of epiphytes and endophytes within plant leaves, stems and roots. Image adapted and modified from Walker et al. (2020).

Plant-associated microorganisms that thrive within the roots, stems, and leaves of plants are termed endophytes. The location of endophytes within the plant compared to epiphytes (microorganisms occurring on the plant exterior) is shown in Figure 2. Endophytic diversity and population are highly variable between plant species and depend on components such as host developmental stage, species, and environmental conditions as well as their lifestyle classification (Surjit and Rupa, 2014). Systemic endophytes have long-term mutualistic associations with plants because they interact and evolve with host plants over many generations thus forming part of the core plant endobiome. In contrast, non-systemic endophytes have short-term associations with plants and their abundance, diversity and association can shift from mutualistic to parasitic depending on the plant development stage as well as biotic and abiotic factors (Orozco-Mosqueda and Santoyo, 2021).

Often, endophytes are bacterial (actinomycetes or mycoplasma) or fungal (yeasts or filamentous) microorganisms. Endophytic fungi colonize the seed, during germination or through penetration of the plant tissues when recognized as a host (Poveda et al., 2021). Examples of endophytic fungi include *Aspergillus*, *Bipolaris*, *Chaetomium*, *Cladosporium*, *Diaporthe*, *Fusarium*, *Alternaria*, *Mucor*, *Nigrospora*, *Paecilomyces*, *Penicillium*, *Piriformospora*, *Porostereum*, *Phoma*, *Trichoderma*, *Ulocladium*, and *Yarrowia* (Orozco-Mosqueda and Santoyo, 2021). Most bacterial endophytes enter plants as rhizobacteria where they perform various plant growth-promoting functions (Pimentel et al., 2011; Nair and Padmavathy, 2014). Gram-positive and gram-negative bacteria, including *Achromobacter*, *Acinetobacter*, *Agrobacterium*, *Bacillus*, *Brevibacterium*, *Microbacterium*, *Pseudomonas*, *Xanthomonas*, were identified from the endosphere (Sun et al., 2013). Various plant growth-promoting bacterial endophytes have been isolated from plants as shown in Table 2. Root nodules, as depicted in Figure 1, are often found on the roots of leguminous plants and consist of endophytes termed nodule-associated bacteria. Tapia-García et al. (2020) isolated 257 nodule-associated bacteria from *Phaseolus vulgaris* plants with the most common plant growth-promoting attributes being indole acetic acid and siderophore production. *Pseudomonas*, *Rhizobium*, *Cupriavidus*, and *Paraburkholderia* were the most abundant bacterial genera isolated from the nodules of the leguminous plants. In another study, endophytes were shown to have a symbiotic relationship with other plant-associated microorganisms. The study concluded that the production of 1-aminocyclopropane-1-carboxylate deaminase by free-living bacteria (such as *Pseudomonas fluorescens*) played a key role in rhizobial nodulation processes by regulating impeding ethylene levels (Nascimento et al., 2019). Endophytes can be beneficial to plants by directly promoting plant growth and development through the metabolism of insoluble nutrients and the production of phytohormones, enzymes, and metabolites. On the other hand, endophytes can indirectly promote plant growth and health by stimulating the capacity of plants to withstand various stresses and their resistance to insects and other pests (Li et al., 2022).

**TABLE 2:** Identified plant growth-promoting bacterial endophytes isolated from various plant species.

Plant species	Plant region	Identified bacterial endophyte	References
Rice (*Oryza sativa*)	Roots	*Stenotrophomonas maltophilia* RR-10	Zhu et al. (2012)
Rice (*Oryza sativa*)	Leaves, stem, and roots	*Klebsiella pneumoniae, Paenibacillus kribbensis, B. aryabhattai, B. megaterium, B. subtilis, Microbacterium binotii, and Microbacterium trichotecenolyticum*	Ji et al. (2014)
Walnut (*Juglans regia*)	Mature fruits	*Bacillus subtilis* HB1310	Zhang Q. et al. (2014)
Rice, sorghum, pearl millet, wheat, and other members of the Poaceae family	Roots	*Achromobacter sp., Acinetobacter sp., Ralstonia sp., Rhizobium sp.*	Patel and Archana (2017)
Cotton (*Gossypium hirsutum* L.)	Stems and roots	*Enterobacter* sp.	Tian et al. (2017)
Blue agave *(Agave tequilana)*	Leaves	*Cronobacter sakazakii, Acinetobacter sp., A. baumannii, A. bereziniae, Enterobacter hormaechei, Klebsiella oxytoca, Bacillus sp. Leuconostoc mesenteroides subsp. mesenteroides, Gluconobacter oxydans, Pseudomonas sp., Enterococcus casseliflavus*	Martínez-Rodríguez et al. (2014)
Tomato *(Solanum lycopersicum)*	N/A	*Pseudomonas sp., Rhizobium sp., Staphylococcus sp., Stenotrophomonas sp., Bacillus sp., Burkholderia sp.*	Patel and Archana (2017)
Greater celandine (*Chelidonium majus*)	Stems	*B. thuringiensis, B. amyloliquefaciens*	Goryluk et al. (2009)
Xaxim (*Dicksonia sellowiana*)	Fern pinnae and rachis	*Gracilibacillus sp., Micrococcus sp., Paenibacillus sp., Stenotrophomonas maltophilia, S. nitroreducens, Amphibacillus sp., B. megaterium, B. pumilus, B. subtilis, and B. thuringiensis*	Fern et al. (2010)

Overall, plant-associated microorganisms play critical roles in agroecosystems by regulating soil fertility, nutrient availability, water sequestration, and plant disease prevention, among other roles (Adeleke et al., 2019). Although the importance of plant-microorganism interactions in agroecosystems has been established, the response of plant-associated microorganisms to emerging nanoparticle applications in agriculture remains unclear.

## 3 Application of nanoparticles in agriculture

Nanotechnology has been described as the understanding and control of matter in the range of 1 to 100 nm (Rajput et al., 2018). Particle dimensions within this range are considered nanoparticles (NPs) (Taghavi et al., 2013). Nanoparticles are distinguished based on their core material (organic or inorganic). Inorganic NPs are further divided into metal (Al, Bi, Co, Cu, Au, Fe, In, Mo, Ni, Si, Ag, Sn, Ti, W, Zn), metal oxide (Al_2_O_3_, CeO_2_, CuO, Cu_2_O, In_2_O_3_, La_2_O_3_, MgO, NiO, SiO_2_, TiO_2_, SnO_2_, ZnO, ZrO_2_), of which Ag, ZnO, TiO2, FeO, and CuO are often utilized and their harmful effects on the activity, diversity, and abundance of flora and fauna are closely observed (Rajput et al., 2018).

Nanoparticles can be used in agriculture as fertilizers or pesticides and are generally regarded as nanofertilizers and nanopesticides, respectively. The use of nanoparticles as nanofertilizers in agriculture has the potential to improve the efficiency of nutrient consumption (Toksha et al., 2021; Ndaba et al., 2022; Rabalao et al., 2022). Additionally, the use of nanoparticles in the form of nanopesticides may protect crops from fungal and bacterial infections (Yadav S. A. et al., 2022). However, the impact of continued use of nanoparticles on plant-associated microorganisms remains unclear. Studies on the effects of nanoparticles on soil and plant microbiomes remain rare, even though microbial communities are important and sensitive determinants of the environmental hazards of nanoparticles (Brookes, 1995; Holden et al., 2014).

Despite the potential benefits of applying nanotechnology to agriculture, some researchers have cautioned and expressed concern about the consequences of nanoparticle applications in agriculture (Khan et al., 2022). Table 3 provides a summary of the benefits and drawbacks of using nanoparticles in agriculture. Nanoparticles are introduced into the agroecosystem via the application of nano-based agriculture amendments as well as the direct release of waste from industries and households (Weir et al., 2012; Sánchez-Quiles and Tovar-Sánchez, 2014). The impact of direct exposure of plants to nanoparticles should not be ignored as they may pose both negative and/or positive effects on soil health as well as crop growth and quality. The factors that influence the effects of nanoparticles include the type and size of the nanoparticle, plant species, nanoparticle concentration, and length of time that the soil/crop was exposed to the nanoparticles (Duan and Li, 2013). In a study done by An et al. (2008) silver nanoparticles boosted ascorbate and chlorophyll in the leaves of asparagus (*Asparagus officinalis* L.). These findings provide examples of the beneficial effects of nanoparticles. In a different study, silica nanoparticles applied to maize seedlings increased seed germination, root and shoot length, photosynthesis, and dry weight (Suriyaprabha et al., 2012).

**TABLE 3:** Advantages and disadvantages of nanoparticle application in agriculture (Sindhu et al., 2020).

**Advantages**	
**Properties**	**Effects**
Facilitate higher nutrient use efficiency	• Small particle size than the pore size of root and leaves leads to more penetration into the plant. • Increase the efficiency with which crop plants absorb nutrients. • Nutrient loss prevention.
Nutrient content and health	• The growth of plant components and metabolic processes like photosynthesis are accelerated by nanofertilizers, increasing yield. • Increased nutrient availability contributes to higher crop quality indicators such as protein, oil content, sugar content, etc. • More readily available nanonutrients protect plants from disease, nutrient shortages, and other biotic and abiotic stresses, resulting in higher yields and higher-quality food products for consumption by humans and other animals.
Slow/controlled release	• For greater uptake by crop plants, nanofertilizers regulate the rate and dosage of encapsulated nutrients and fertilizers. • Increased availability as a result of nutrients’ gradual release. • Extend the real time that nutrients are supplied for.
Reduces loss	• The slower rate of release ensures constant nutrient availability. • Plants can absorb nutrients without wasting them by leaching and/or leaking. • Decrease the need for fertilizers.
Enhance the soil’s quality	• Improve soil quality and water-holding capacity. • Improves microbial activity.
**Disadvantages**	
Transformation of NPs	• Nanomaterials can interact and modify various elements of the environment due to their reactivity. • Nanomaterials may cause toxicity when they interact with soil components.
Accumulation of NPs	• Nano-fertilizers can build up in plant tissues, which can limit growth, produce reactive oxygen species, and cause cell death. • May build up in food components and, when consumed, may have negative effects on human health.
Safety concerns for farm workers	• Reactivity and unpredictability of Nano-materials have prompted safety issues for personnel who may become exposed during their fabrication and deployment in the field.

On the other hand, some reports on metal nanoparticles (MNPs) suggest negative impacts on the growth and physiology of internationally significant crops like maize (*Zea may* L.), wheat (*Triticum aestivum*), rice (*Oryza sativa* L.) and soybean (Dimkpa et al., 2012; Nair and Chung, 2014; Thuesombat et al., 2014). The toxic effects of nanoparticle application on crops are both physical and physiological, and examples include a reduction in fruit yield, plant growth, and biomass. Nanoparticles may also cause indirect toxicity to plants by damaging plant roots, enhancing uptake of contaminants by plants, and by altering plant-associated microbial communities (Anjum et al., 2013; Ge et al., 2014). The mechanisms by which nanoparticles interact and impact plant associated microorganisms, following their application, is discussed in the following sections.

### 3.1 Interaction of nanoparticles with plant-associated microorganisms

The potential use of nanoparticles as nanofertilizers and nanopesticides for precision and sustainable agriculture is still in its infancy and is currently under rigorous investigation (Zulfiqar et al., 2019; Hazarika et al., 2022; Zain et al., 2024). The application of nanofertilizers and nanopesticides may impact various plant growth characteristics (such as seed germination, root and shoot growth, chlorophyll content, photosynthesis, flowering, fruit formation, as well as crop yield), depending on the plant’s genetic makeup, soil and plant microbiology, soil nutrients (macronutrients and micronutrients), soil pH, moisture, and other environmental factors (Juo and Franzluebbers, 2003; Bratovcic et al., 2021; Okey-Onyesolu et al., 2021). Nanoparticles introduced in soil and plants could directly or indirectly affect the type of microorganisms present and alter their functions (Mosquera et al., 2018; Kibbey and Strevett, 2019).

The effects of nanoparticles on plant-associated microbial communities are highly dependent on the plant type, nanoparticle type (physical characteristics and chemical composition), soil properties (i.e., clay and organic matter content), as well as soil physicochemical characteristics (texture, organic matter content, pH, etc.) (Kumar et al., 2018; Kibbey and Strevett, 2019; Peng et al., 2020). In the rhizosphere, plants release a variety of exudates that promote microbial growth. Meanwhile, microorganisms work in concert with plant roots to support plant growth by facilitating a variety of nutrient cycles (Zhang N. et al., 2014). The presence of nanoparticles in soil dramatically affects the microbial communities in the rhizosphere, plant exudates, and extracellular materials produced by the microorganisms (Gao et al., 2018). Additionally, nanoparticles can enter the plant directly through root and stomata pores on leaf surfaces, with diameters ranging from a few tens of nanometers to a few hundred (Carpita et al., 1979; Eichert and Goldbach, 2008; Eichert et al., 2008). Subsequently, nanoparticles are transported by plasmodesmata from cell to cell within the plant, where they affect various physiological functions as well as plant endophytes (Zambryski, 2004).

Nanoparticle-microbe interactions within the plant and soil play a significant role in disease management and subsequent plant improvement. However, this is influenced by either negative or positive nanoparticle effects as antimicrobial agents or microbial growth promoters, respectively. The mechanism in which nanoparticles hinder the development of various microorganisms involves the release of metal ions that interact with cellular components through various pathways. These pathways include generation of reactive oxygen species (ROS), formation of pores in the cell membrane, damage to cell walls, DNA damage, and cell cycle arrest. Ultimately, all these lead to the inhibition of cell growth and in some cases, phytopathogen inhibition (Singh et al., 2019).

While many studies may have focused on nanoparticle mechanisms as antimicrobials, it has also been shown that nanoparticles can play a positive role on microbial metabolism and functions. The beneficial nanoparticle-microbe interactions include nanoparticles’ high bioavailability due to increased specific surface areas. This helps in nutrient uptake by the microbes as nanoparticles provide microorganisms with essential nutrients that stimulate growth and metabolic activity. Nanoparticles can also act as nano-tools for electron transfer, chemotaxis, and storage units (Mansor and Xu, 2020).

Learning about the mechanisms in which microorganisms interact with nanoparticles might help in the development of nanomaterials that are safe for the environment. This can include development of green synthesis approach for nanoparticle production. Overall, the use of nanoparticles as agricultural amendments requires further investigation as it may directly or indirectly affect plant growth by influencing plant-associated microorganisms.

#### 3.1.1 Impact of nanofertilizer on plant-associated microorganisms

Nanoparticles, when used in the form of nanofertilizer, have been proven to enhance crop growth and quality (Merghany et al., 2019; Babu et al., 2022). Unlike bulk chemical fertilizers, which are required in high doses, nanofertilizers can be applied in relatively smaller quantities. Applying a lower dosage of nanofertilizer can minimize the potential for nutrient loss through leaching and volatilization, and thereby improves nutrient use efficiency (Raliya et al., 2018).

Three factors – intrinsic, extrinsic, and mode of administration – affect the efficiency of nanofertilizers. Nano-formulation techniques, particle size, and surface coating are examples of intrinsic variables. While extrinsic factors include soil texture, depth, pH, temperature, organic matter, and microbial activity (Zulfiqar et al., 2019). Moreover, the mechanism of delivery through plant roots or leaves (foliar) has a considerable impact on the uptake, behavior, and bioavailability of nanofertilizers (Mahil and Kumar, 2019). Due to their interaction with organic materials in the soil, nanofertilizers may change the soil surface chemistry, which could have an impact on plants and microorganisms. On the other hand, microorganisms and their actions can potentially alter how nanoparticles behave (Frenk et al., 2013; Zulfiqar et al., 2019; Toksha et al., 2021).

In a study conducted by Kaur et al. (2022), the effect of titanium dioxide (TiO_2_) NPs on the soil rhizosphere of mung bean crop was evaluated. The TiO_2_ NPs were shown to stimulate growth of soil microflora (N-fixers and ammonia oxidizers) as well as increase enzymatic activity for dehydrogenase, phosphatase, protease, urease, and catalase at low concentrations (1.0, 2.5, 5.0, and 10.0 mg/L) compared to the higher concentration (20 mg/L). In addition, the nitrate-N content increased with days after treatment and TiO_2_ NP concentration. An experiment conducted by Helal et al. (2023) demonstrated that the tomato plant (*Lycopersicon Esculentum* L.) treated with a controlled-release nano-urea (CRU) fertilizer showed better plant growth, yield, and fruit quality compared to the conventional fertilizers. In another study, an increase in nutritional value of spinach (*Spinacia oleracea*) after treatment with ZnO NPs (500 and 1,000 ppm) was indicated by higher values of protein and dietary fiber, as well as overall leaf quality (width, length, color, and surface area) (Revanappa and Pramod, 2015). Table 4 highlights some of the impacts of different nanofertilizers on microbial processes and related microorganisms in plants.

**TABLE 4:** Impact of nanofertilizers on microbial functions and related microorganisms in plants.

Nanomaterial	Plant name	Effect on microorganisms	Effect on microbial function	Effect on the plant	References
Metallic silver (Ag)	*Cucumis sativus*	Increased growth-promoting bacterial activity	Improved carbon, nitrogen, and other biogeochemical cycles	An increase in the length of the roots and shoots as well as biochemical indicators like proline, protein, and antioxidants	Nawaz and Bano (2019)
Titanium dioxide (TiO2)	*Triticum aestivum*	Increased actinobacterial and planctomycete abundance	Efficiency of nitrogen fixation increased	Improvement in phenotypic characteristics	Moll et al. (2017)
ZnO NPs	*Phoenix dactylifera*	Number of fungal and bacterial cultivable heterotrophic colony-forming units reduced significantly	Reduction in carbon and nitrogen mineralization efficiency	Decrease in dissolved organic carbon and mineral nitrogen	Rashid et al. (2017)
Iron oxide (FeO)	*Zea mays*	An increase in *Bradyrhizobium* and ammonia-oxidizing bacteria activity	Improved nitrification	Improved plant growth and yield.	He et al. (2016)
Iron oxide (Fe_3_O_4_)	*Triticum aestivum*	Increased actinobacteria and planctomyces population	Improved nitrogen fixation efficiency	Improvement in phenotypic characteristics	Zhang et al. (2020)
Pristine and sulfidized ZnO NPs	*Glycine max*	Significant effects on bacterial communities	Drastic impact on carbon and nitrogen metabolism	Overexposure to zinc may have an impact on the development and growth of soybeans	Chen et al. (2023)
Zinc oxide (ZnO)	*Lactuca sativa*	Increased abundance of cyanobacteria, bacteria, and protozoa	Enhancement of organic matter decomposition and nitrogen fixing	Fresh biomass and net photosynthetic rate both increased by 6.2%	Xu et al. (2018)
Cu and Zn NPs	*Raphanus sativus*	Reduced Azotobacter genus abundance in the soil	Decrease in catalase and dehydrogenase activities	Decrease in germination and roots length	Kolesnikov et al. (2019)
Silica	*Zea mays*	P solubilizing and nitrogen-fixing bacteria were more abundant, but silicate-solubilizing bacteria were less abundant	N/A	Increased germination and absorption of silica	Rangaraj et al. (2014)
High dose of ZnO NPs	*Medicago sativa*	Reduction in the quantity of bacteroids and in the diversity and relative abundance of soil microorganisms	Decreased nitrogen-fixing ability	Decrease in root nodules and plant biomass	Sun et al. (2022)

Despite the many benefits of nanofertilizers, the antibacterial potential of nanoparticles in general has also received substantial attention (Nath et al., 2008; Ramírez Aguirre et al., 2020; Thakral et al., 2021). The applied nanofertilizers may inadvertently have negative impacts on the beneficial microbial populations in the soil and on plants. The concentration and identity of nanoparticles, soil type, pH, and biological factors including root exudates and microbial diversity all have a significant impact on how nanoparticles affect the soil. A study conducted by Xu et al. (2015) investigating the effect of CuO NPs on soil microbes in flooded paddy soil reported CuO NPs (500 and 1,000 mg/kg) to have a negative impact on the soil microbes as was indicated by a significant decrease in microbial biomass and decrease in enzyme activity for urease, phosphatases, and dehydrogenase. The application of silver nanoparticles (Ag NPs) at 100 mg/kg significantly increased the soil pH and altered bacterial groups associated with carbon, nitrogen, and phosphorus cycling both in the absence or presence of cucumber (*Cucumis sativus*) plants (Zhang et al., 2020). Metal oxide nanoparticles, namely ZnO and CeO_2_, were observed to inhibit enzymatic activity and reduced the numbers of K-solubilizing and P-solubilizing bacteria as well as soil Azotobacter (Zhang et al., 2018; Kaur et al., 2022). In another study, the activity of soil dehydrogenase was demonstrated to be adversely affected by high quantities of nanoparticles (García-Gómez et al., 2018). Dehydrogenase activity directly correlates with soil microbial biomass, and plays a significant role in the oxidation of organic materials. Therefore, the microbial biomass was impacted by the dose of nanoparticles applied (García-Gómez et al., 2018). Another study by Shah et al. (2014) examining the response of the soil microbial community to nanoparticle application showed that silver nanomaterial caused changes in the microbial community structure, however, zinc oxide and zero-valent copper oxide did not significantly alter the structure of the microbial community.

#### 3.1.2 Impact of nanopesticides on plant-associated microorganisms

Plant diseases and insect pests are effectively managed in agriculture by the application of pesticides. However, the high concentrations of chemical components applied per hectare has given rise to several issues, including environmental deterioration, pest resistance, bioaccumulation, and health risks (Yadav J. et al., 2022). Due to microbial activity, air drift, soil leaching, degradation processes including photolysis and hydrolysis, amongst other factors, more than 90% of the pesticides that are applied are lost. It is only a small amount of the remaining 10% that eventually reaches the target site (Yadav J. et al., 2022). This necessitates repeated application which eventually results in high costs and environmental pollution. Moreover, certain pesticides have been shown to have adverse effects on human health such as cancer, birth defects, reproductive defect, neurological and developmental impairment, immunotoxicity, and disruption of the endocrine system, when ingested through the consumption of pesticide-contaminated food (Toksha et al., 2021).

Although some environment-specific nanopesticides are on the market (Smith et al., 2008), nano-formulations with effective delivery mechanisms which result in application of modest amounts of nanopesticides are required. Nanopesticides provide innovative strategies for delivering the active ingredient of pesticides to the target site (Ahmed et al., 2023). Slow-releasing qualities, enhanced stability, permeability, solubility, and specificity are all features of nano-encapsulated pesticide formulations (Narayanan et al., 2017). They are specifically created to make the active ingredient (AI) more soluble and release it at the target site in a controlled manner. Due to this, only a small amount of the AI needs to be applied for it to be effective for an extended period of time (Oliveira et al., 2019).

Nanopesticides are classified into two types. Type 1 nanopesticides are metal-based, whereas Type 2 materials contain AIs that are enclosed by nanocarriers, such as polymers, clays, and zein nanoparticles. The most prevalent analytes for Type 1 nanopesticides are Ag-, Ti-, and Cu-based nanomaterials (NMs). These nanopesticides can suppress a variety of plant pathogens, including fungal (such as *Candida* and *Fusarium*), as well as bacterial (such as Escherichia coli and Staphylococcus) (Wang et al., 2022). If properly applied, nanopesticides could increase crop output, food safety, and nutritional value. For several plants treated with Type 1 nanopesticides (such as Ag-, Ti-, Cu-, and Zn-based NMs), improvements in the concentration of sugar, fatty acids, chlorophyll, carotenes, and important elements (such as P, K, Ca, Mg, S, Fe, Si, Mn, and Zn) have been documented (Gomez et al., 2021; Ma et al., 2021; Rawat et al., 2021; Shang et al., 2021; Yadav J. et al., 2022). Suppression of pathogenic activity is one of the factors contributing to these enhancements.

The abundance, structure, and network functioning of the plant-associated microbiome, which includes archaea, bacteria, and fungi, can be changed by adding metal-based nanopesticides to soil and plant. This in turn may change the bioavailability and recycling of macronutrients (such as C, N, P, and S). More importantly, in order to fully utilize nanopesticides, it is necessary to comprehend how they interact with nutrients, soil, plant-associated microbiota, and other factors. Nanopesticides have obvious pesticidal activity and as such can exhibit toxicity toward non-target organisms. Studies show that, in comparison to their non-nanoscale equivalents, nanopesticides are 43.1% less toxic (Wang et al., 2020). This is primarily due to their AI delivery system, which is target-specific, and thereby minimizes the exposure to non-target organisms.

Cu(OH)_2_ nanopesticides applied to target soil agroecosystems for 365 days, had only minor negative effects on non-target wetland systems and the bacterial and fungal communities that live there (Carley et al., 2020). However, a few studies have shown negative impacts related to nanopesticide exposure. Zhai et al. (2020) showed that long-term exposure to high concentrations of atrazine-containing nanopesticides (NPATZs) dramatically reduced the metabolic capability of bacterial communities in the rhizosphere and changed the makeup of those communities in comparison to conventional ATZ. An investigation into the long-term (117 days) effects of Ag nanopesticides (100 mg/kg) on the microbiome of the maize rhizosphere revealed negative effects on microbial diversity, the nitrogen cycle, and crop output (Sillen et al., 2020). Low concentrations (0.5, 1.0, and 2.0 mg/g) of zinc oxide (ZnO) applied directly to soil enhanced the relative abundance of the essential bacterial group *Bacillus* in comparison to the control, but the higher concentrations had harmful effects on the bacterial population (You et al., 2018). A study by Zhao et al. (2017), discovered that exposure of spinach to Cu(OH)_2_ nanopesticide resulted in a significant reduction in antioxidant or defence-associated metabolites such as ascorbic acid, α-tocopherol, threonic acid, β sitosterol, 4-hydroxybutyric acid, ferulic acid, and total phenolics (Peixoto et al., 2021). Another study showed that captan@ZnO35-45 nm and captan@SiO2 20–30nm nanofungicides influenced soil microorganisms by altering numerous microbial characteristics (Sułowicz et al., 2023).

Overall, literature suggests that nanopesticides may be more effective, resilient, and sustainable than their traditional analogues, with fewer negative environmental effects. However, future research is required to comprehend the effects of realistic nanopesticide doses on the rhizosphere microbiota, crop yield, and agroecosystem health in field settings.

## 4 Conclusion and future prospects

Based on the literature investigated in this review, the use of nanoparticles as nanofertilizers or nanopesticides were shown to have both beneficial and negative effects on plant-associated microbial populations as well as crop and soil properties. The implications of exposing agricultural environments to nanoparticles can therefore be beneficial or detrimental with respect to the health of agroecosystems and as a result of downstream consumption of crops. Moreover, the environmental risk assessment of nanoparticles is in its infancy. Thus, further research studies investigating the impact of different types and doses of nanoparticles, applied under varying environmental conditions, on microbial communities and function, especially long-term, are necessary. Despite the rise in the manufacturing of nanoparticles for agricultural applications, the majority of risk assessment testing is conducted *in-vitro* using cells rather than animals as test subjects. Therefore, more research on soil and human health implications is necessary due to the ambiguities surrounding the negative consequences of nanoparticle applications. In the context of impact of nanofertilizers and nanopesticides, plant-associated microorganisms indicative of healthy/unhealthy crops and soils should be employed as sensitive biomarkers to assess the environmental risk of these nanomaterials. Moreover, prospective studies should investigate the impact of nano-based agricultural amendments under different conditions such as crop type, soil properties and microbial community dynamics for the compilation of a database that can provide a case-by-case basis for precision agricultural practices incorporating the utilization of nanoparticles.

## Author contributions

KM: Conceptualization, Writing—original draft, Writing—review and editing. BN: Conceptualization, Funding acquisition, Project administration, Supervision, Writing—review and editing. AR: Funding acquisition, Supervision, Writing—review and editing. HR: Writing—review and editing. RA: conceptualization, Funding acquisition, Supervision, Writing—review and editing.
